# Phosphoric Acid for Foul Ulcers

**Published:** 1898-09

**Authors:** 


Phosphoric Acid for Foul Ulcers. A 10 per cent. solution of pure phosphoric acid in distilled water is highly recommended in human practice as an application for foul ulcers of a scrofulous or tuberculous nature. The fluid can also be used hypodermatically in hypertrophied glands.

